# Pre-vaccination SARS-CoV-2 seroprevalence among staff and residents of nursing homes in Flanders (Belgium) in fall 2020

**DOI:** 10.1017/S095026882200036X

**Published:** 2022-03-02

**Authors:** Heidi Janssens, Stefan Heytens, Eline Meyers, Ellen De Schepper, An De Sutter, Brecht Devleesschauwer, Asangwing Formukong, Sara Keirse, Elizaveta Padalko, Tom Geens, Piet Cools

**Affiliations:** 1 Research and Analytics, Liantis, Belgium; 2 Department of Public Health and Primary Care, Faculty of Medicine and Health Sciences, Ghent University, Ghent, Belgium; 3 Department of Diagnostic Sciences, Faculty of Medicine and Health Sciences, Ghent University, Ghent, Belgium; 4 Biostatistics, Faculty of Medicine and Health Sciences, Ghent University, Ghent, Belgium; 5 Department of Epidemiology and Public Health, Sciensano, Brussels, Belgium; 6 Department of Veterinary Public Health and Food Safety, Faculty of Veterinary Sciences, Ghent University, Merelbeke, Belgium; 7 Department of Medical Microbiology, Ghent University Hospital, Ghent, Belgium

**Keywords:** SARS-CoV-2, Nursing homes, serology, Belgium

## Abstract

Seroprevalence of severe acute respiratory syndrome coronavirus 2 (SARS-CoV-2) IgG antibodies, using dried blood spots, was determined in October–November 2020, among residents and staff randomly selected from 20 nursing homes (NH) geographically distributed in Flanders, Belgium. Sociodemographic and medical data [including coronavirus disease 2019 (COVID-19) symptoms and results of RT-PCR tests] were retrieved using questionnaires. The overall seroprevalence was 17.1% [95% confidence interval (CI) 14.9–19.5], with 18.9% (95% CI 15.9–22.2) of the residents and 14.9% (95% CI 11.9–18.4) of the staff having antibodies, which was higher than the seroprevalence in blood donors. The seroprevalence in the 20 NH varied between 0.0% and 45.0%. Fourteen per cent of the staff with antibodies, reported no typical COVID-19 symptoms, while in residents, 51.0% of those with antibodies had no symptoms. The generalised mixed effect model showed a positive association between COVID-19 symptoms and positive serology, but this relation was weaker in residents compared to staff. This study shows that NH are more affected by SARS-CoV-2 than the general population. The large variation between NH, suggests that some risk factors for the spread among residents and staff may be related to the NH. Further, the results suggest that infected people, without the typical COVID-19 symptoms, might play a role in outbreaks.

## Introduction

Worldwide, Belgium was one of the countries with the highest number of coronavirus disease 2019 (COVID-19) deaths per capita [1]. About 60% of these deaths were residents from nursing homes (NH) [2]. Also, in Europe, the WHO estimated that at the end of June 2020, about half of the COVID-19 deaths were NH residents [3].

So far, seroprevalence data have been published from the general Belgian population, primary health care workers and more recently also from school children and staff [4]. However, the reports on seroprevalence results, based on samples before 2021 (before the start of the vaccination campaign), are limited. The available results [4, 5], revealed that, the percentage of blood donors (as a proxy for the general Belgian population) with severe acute respiratory syndrome coronavirus 2 (SARS-CoV-2) antibodies was increasing from 5.0% (end of April 2020) up to 9.2% by the end of October 2020. For hospital staff [4, 6, 7], the seroprevalence was varying between 7.7% (end of April 2020) and 10.8% (end of October 2020).

Also, to our knowledge, seroprevalence data from a representative sample of NH in Belgium, before the start of the vaccination campaign, are still lacking. Moreover, it is important not only to focus on residents [8], but also to assess the seroprevalence of the staff from the NH. Additionally, it should be noted that, according to several reports, asymptomatic and presymptomatic people played an important role in the transmission of COVID-19 in long-term care facilities [9]. Consequently, seroprevalence numbers will not only provide a measure of cumulative incidence of SARS-CoV-2 infections, but also provide more insight into the role of asymptomatic or presymptomatic people in the transmission of the virus within NH.

Therefore, the main goal of the current study is to assess the seroprevalence of SARS-CoV-2 in both staff and residents from NH in Flanders, Belgium.

## Methods

### Ethical considerations

Ethical approval was obtained by the Ethical Committee of the Ghent University Hospital (reference number BC-07665). The NH management informed residents and their families, and staff on the study objectives and sampling procedures. Residents (and/or family of residents) and staff who agreed to participate in the study signed an informed consent form. A confidential counsellor (a family member or a nurse after approval by the family) signed for participants who were incapable to sign the consent form, such as residents with dementia.

### Study design

The current manuscript is the first report of the overarching SARS-CoV-2 Liantis study. This cross-sectional study was designed to (i) assess the seroprevalence in Belgian NH after the first wave and (ii) identify risk factors at both the individual (residents and staff) and NH level for SARS-CoV-2 infection in the first SARS-CoV-2 wave in Belgium. The end of the first wave of the epidemic in Belgium was defined retrospectively by the number of confirmed cases which was at its lowest level by 22 June 2020 [10]. Retrospectively, the start of the second wave, which was determined by the evolution of both the number of new cases and the number of hospitalisations remaining positive throughout the week, was set on 31 August 2020 [10]. Between 19 October and 13 November 2020, the SARS-CoV-2 Liantis study tested NH residents and staff for the presence of SARS-CoV-2 IgG antibodies and collected demographic, behavioural, clinical (including results of previous SARS-CoV-2 RT-PCR tests) and NH-specific data by means of questionnaires. Individual and NH-specific risk factors for SARS-CoV-2 seroprevalence after the first wave will be reported elsewhere. In the current manuscript, we report the seroprevalence of SARS-CoV-2 in residents and staff.

### Sample size

To assess both study objectives of the overarching SARS-CoV-2 Liantis study (seroprevalence and identification of risk factors), the clustering of participants within NH was taken into account for the sample size calculation. To assess the seroprevalence in NH in Belgium, a sample of 381 participants (residents and staff members) would be sufficient to estimate the seroprevalence of 50% with a half-width of 5%, using a 95% Wilson score confidence interval (CI). However, to cover the purpose of identifying risk factors at individual and NH level, calculations demonstrated that 80 NH, with each 60 participants, across Belgium, were needed. To anticipate non-response, 100 NH were initially selected.

### Selection and recruitment of the NH

The NH were selected from a database of Liantis, a Belgian external occupational health service. A subset of 210 NH employing at least ten staff members was used. In order to obtain a representative geographical sample of the Belgian NH, the 100 NH were chosen according to the true proportion of NH per province in Belgium, based on data available in the Crossroads Bank for Enterprises [11].

This procedure resulted in the desired number of 56 NH, situated in the five provinces of the Flemish region and the Brussels capital region, and 44 NH in the five provinces of the Walloon region. The management from each selected NH was contacted to explain the study and request for participation. In case of refusal, another (random sampled) NH in the same province was contacted.

Unfortunately, the rapid progression of the 2nd wave of COVID-19 in Belgium [12], which initially affected particularly the Walloon region in Belgium around October 15th, forced us to stop sampling prematurely even before any sampling was carried out in Wallonia. Since two study goals (not reported here) were to address individual and NH-specific risk factors that occurred during the first wave, further sampling would bias the relationship between these risk factors and prevalence. In this way, we found 20 NH from the 56 selected NH, in the Flemish and Brussels capital region, willing to participate.

### Selection of the staff and residents

In every included NH, *n* residents and 60-*n* staff were randomly selected using an online tool specifically developed for this study. The number of selected residents and staff in each NH reflected the proportion of residents and staff in that NH. In one smaller nursing home, the selection was limited to 45 residents and staff due to the limited number of staff. For the staff members, there were no exclusion criteria. For the residents, those living in assisted living facilities were excluded. For each staff member or resident who refused to participate, an additional randomly selected staff member or resident was invited to participate.

### SARS-CoV-2 serology

To assess the presence of SARS-CoV-2-specific IgG antibodies, capillary blood was collected via a finger prick from each participant on protein saver cards and air-dried (i.e., dried blood spots, DBS) as previously described [13]. A pilot study showed that the sensitivities and specificities of DBS were at least 95% and 97%, respectively, depending on population (residents or staff) or DBS card type [13]. DBS were sent to the Laboratory Bacteriology Research (LBR, Department Diagnostic Sciences, Faculty of Medicine and Health Sciences, Ghent University) and stored at four °C in an airtight container of low humidity and analysed within a period of 5 days. The DBS were analysed for the presence of anti-spike (anti-S) IgG antibodies by means of ELISA (EUROIMMUN, PerkinElmer Health Sciences Inc., Lübeck, Germany) as described previously [13]. Participants were classified as seronegative or seropositive according to the antibody optical density results of the ELISA (<0.8 or ≥0.8 respectively).

### Questionnaires

Two individual (one for staff and one for residents) and one NH-specific questionnaire were designed in LimeSurvey [14] and applied after review and testing by a number of the present co-authors and volunteers from the target population. Basically, the individual questionnaires asked about sociodemographics, COVID-19 symptoms, laboratory test results, SARS-CoV-2 exposure and prevention measures. To question the typical Covid-19 symptoms, we presented the symptoms defined in Sciensano's case definition and asked whether the worker/resident has suffered from them since February 2020 [15]. The NH questionnaire asked about the NH SARS-CoV-2 epidemiological history, NH-specific demographics, potential risk factors related to the building and infrastructure, the overall frailty of the residents and the measures that were taken at several stages during the pandemic. Questionnaires were available in Dutch and French and after receiving a unique code, participants got access to the digital version [14]. Residents were assisted by a nurse or general practitioner to fill in the questionnaire. The questionnaires are available from the corresponding author upon request.

### Statistical analysis

LimeSurvey online questionnaire responses were exported as R data files. Statistical analyses were performed using R 3.6.0 [16, 17].

Prevalences (and 95% CIs) were reported as frequencies of positive SARS-CoV-2 antibody tests proportional to the total sample size, using a one-sample proportion test with continuity correction. Results were compared with the prevalence of the Belgian population at the end of October (9.2%). *χ*^2^ or Fisher exact and *t*-tests were applied to assess differences between respectively categorical and continuous variables. After investigating the relation between the variable ‘staff versus resident’ and serology result, we additionally explored the relation between the serology and the self-reported RT-PCR results and self-reported manifestation of COVID-19 symptoms. Taking into account the clustered structure of the data, generalised linear mixed-effects models for a binary outcome using a logit link were applied. In each model, NH were considered as a random effect and the variables gender, staff/resident, COVID-19 symptoms, and self-reported RT-PCR test results as fixed effects. A backward procedure was applied, removing non-significant variables and interaction terms (*P* > 0.05) from the model.

## Results

### Study population

There was no significant difference (Fisher exact test, *P* = 0.356) between the geographical distribution of these 20 NH within the Flemish and Brussels Capital Region and the data available in the Crossroads Bank for Enterprises [11]. In Table 1, a number of characteristics of the selected NH are displayed.

**Table 1.** Characteristics of the twenty included nursing homes (NH)

Geographical location^a^ of NH	Number (%) of NH
Flemish region	18 (90)
*Antwerp*	*2 (10)*
*East-Flanders*	*5 (25)*
*Limburg*	*1 (5)*
*Flemish-Brabant*	*4 (20)*
*West-Flanders*	*6 (30)*
Brussels Capital Region	2 (10)
Type of NH	Number (%) of NH
Public	4 (20)
Private	3 (15)
Non profit	13 (65)
Urbanisation of geographical location of NH	Number (%) of NH
City	4 (20)
Periphery	12 (60)
Rural area	4 (20)
Size of NH	Median + /− IQR (min-max) of beds^b^
	117.5 + /− 48.0 (50–300)
**COVID-19 related mortality (both proved and suspected deaths), since start of the epidemic**	**Median + /− IQR (min-max),** expressed as a ratio (in %) of the number of deaths over the number of residents^c^
	2.5 + /−7.7 (0–17.4)

IQR, interquartile range. The bold texts are representing the different variables.

a Geographical location is divided in two regions. For the Flemish region, a further division in provinces (italic text) is given.

b All beds in the NH, excluding those of the assisted living facilities.

c Includes data from 19 of the 20 NH.

An overview of the number of participants with respectively questionnaire and serology results is displayed in Figure one. We included a total of 1185 participants – 508 staff members and 677 residents – in the study. From 460 staff members and 601 residents, complete questionnaire data were received, corresponding to a response rate of 89.5%. From 478 staff members and 615 residents, a valid serological test result was obtained, which corresponds to 92.2% of the total study population. This resulted in 454 staff members and 577 residents with valid serology results and complete questionnaire data (87.0%). Seroprevalence was reported based on all available serology test results. The correlation of serological results with sociodemographic variables was based on complete cases for serology results and questionnaire data and modelling was assessed on the subset of 998 participants, where also a self-reported RT-PCR test result was available.

**Fig. 1.**
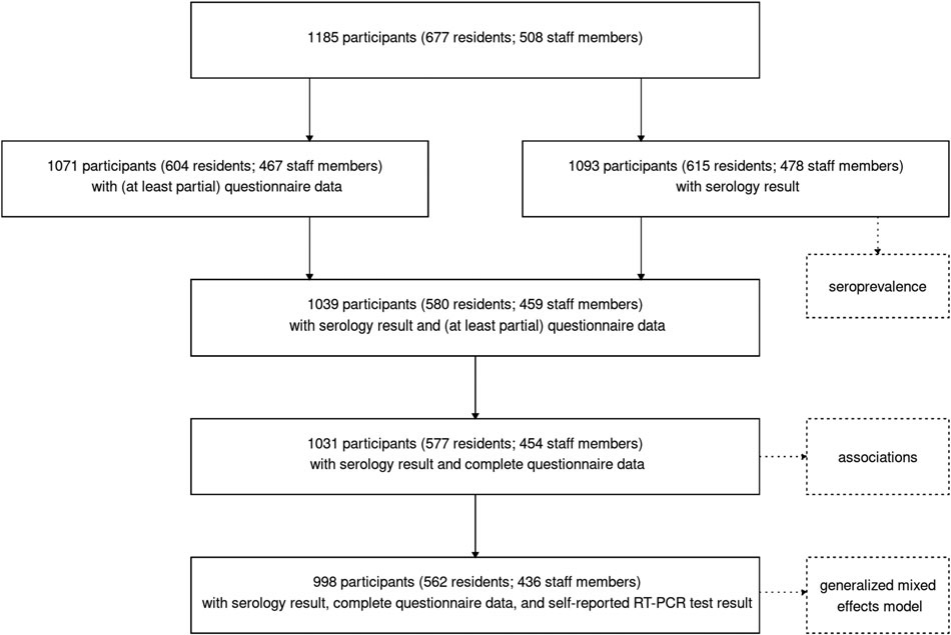
Number of included participants with a questionnaire (including self-reported RT-PCR results) and serology results.

In Table 2, the demographics and clinical characteristics of the participants are summarised. The study population was predominantly female (70.9%). The mean age of the staff was 41.6 years, while the mean age of residents was 85.5 years. The majority of staff (79.5%) consisted of nurses, caregivers and paramedics.

**Table 2.** Characteristics of participants, stratified for residents and staff members, based on answers on the individual questionnaires

	Residents (*N* *=* *577*)	Staff (*N* *=* *454*)
Gender	*n* (%)	*n* (%)
Female	409 (70.9)	397 (87.4)
Male	168 (29.1)	57(12.6)
Age (years)	Mean + /− s.d.	Mean + /− s.d.
	85.5 + /− 7.81	41.6 + /− 11.79
Job type		*n* (%) ^a^
Nurse or caregiver		196 (43.3)
Cleaning or logistics		61 (13.5)
Kitchen aid or cook		6 (1.3)
Paramedic function or animator		164 (36.2)
Technical support		4 (0.9)
Administration		11 (2.4)
Management		11 (2.4)

s.d., standard deviation.

a Includes data from 453 of the 454 staff members.

### Prevalence

The overall seroprevalence was 17.1% (95% CI 14.9–19.5), with 18.9% (95% CI 15.9–22.2) of the residents and 14.9% (95% CI 11.9–18.4) of the staff having antibodies (Table 3).

**Table 3.** Number and percentage of seropositive/seronegative residents and staff members

	Residents: *n* (%)	Staff: *n* (%)	*P*-value (*χ*^2^ test)
Seropositive^a^	*N* *=* *615*	*N* *=* *478*	
No	499 (81.1)	407 (85.1)	0.081
Yes	116 (18.9)	71 (14.9)	
Self-reported COVID-19 symptoms^b^	*N* *=* *577*	*N* *=* *454*	
No	486 (84.2)	360 (79.3)	*P* < 0.001
Yes	91 (15.8)	94 (20.7)	
Minimum one self-reported positive RT-PCR test^b^	*N* *=* *562*	*N* *=* *436*	
No	472 (84.0)	371 (85.1)	0.696
Yes	90 (16.0)	65 (14.9)	

Results of the answers on questions related to RT-PCR test and COVID-19 symptoms, stratified for residents and staff members.

a Results are based on participants with serology results.

b Results are based on participants with complete questionnaire data.

The seroprevalence per NH varied between 0% and 45.0%. In four of the 20 NH, none of the participants had antibodies. In three of the NH, the seroprevalence was zero for staff only. In these NH, the seroprevalence was low (2.8%, 4.9% and 9.4%) in residents. Vice versa, in one NH, none of the residents were found to be seropositive. Here, seroprevalence in staff was also low (3.8%). In 11 of the 20 NH, the seroprevalence was higher than 9.2%, the estimated seroprevalence for the Belgian population at the end of October 2020 [4].

Of those who were tested by means of RT-PCR, 90 residents (16.0%) and 65 positive workers (14.9%) had at least one positive test (Table 3). Almost 21% of staff reported having had symptoms typically for COVID-19, while this was the case in about 16% of the residents.

### Associations between SARS-CoV-2 seropositivity, self-reported RT-PCR test results and self-reported COVID-19 symptoms

The proportion of seropositive results in relation to self-reported RT-PCR test results and COVID-19 symptoms is shown in Figure three. A total of 6.3% ([17 + 36]/[17 + 354 + 436 + 36]) of the participants who reported a negative RT-PCR, had a positive serology (staff: 4.6% (17/[17 + 354]); residents: 7.6% (36/[436 + 36])) (Figure three, upper panel). Almost 30% (29.7% ([20 + 23]/[45 + 20 + 23 + 67])) of participants reporting at least one positive PCR (staff: 30.8% (20/[45 + 20]) and residents: 25.6% (23/[23 + 67])), had no detectable antibodies (Figure three, upper panel).

A total of 14.3% (9/[9 + 54]) of the staff with antibodies, reported they had not had any typical symptoms compatible with COVID-19 disease, while in residents, about 51.0% (53/[53 + 51]) of those with antibodies reported they had not had the typical symptoms (Figure three, lower panel).

Since large differences in seroprevalence between NH were observed (see also Figure two), the relation between the serology results and the variable RT-PCR results, COVID-19 symptoms and resident/staff was modelled using a generalised linear mixed-effects model. The intraclass correlation coefficient (ICC) indeed demonstrated that about 44.3% of the variance in serology result is situated at the NH level.

**Fig. 2.**
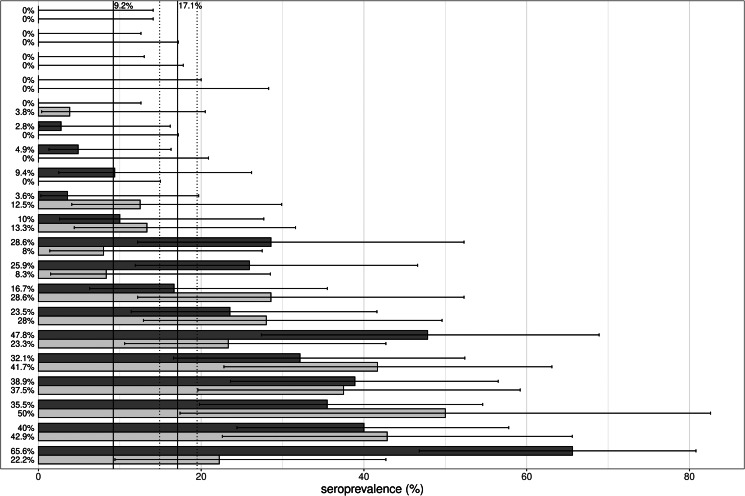
Graphical presentation of the estimates of seroprevalence (%) with 95% CIs, stratified per nursing home, displayed separately for staff members (light grey) and residents (dark grey). The black horizontal lines represent the 95% CI of the seroprevalence estimates. The vertical black lines indicate the seroprevalence of the general Belgian population at the end of October (9.2%) [4] and the overall seroprevalence (17.1%) with 95% CI (dotted lines).

**Fig. 3.**
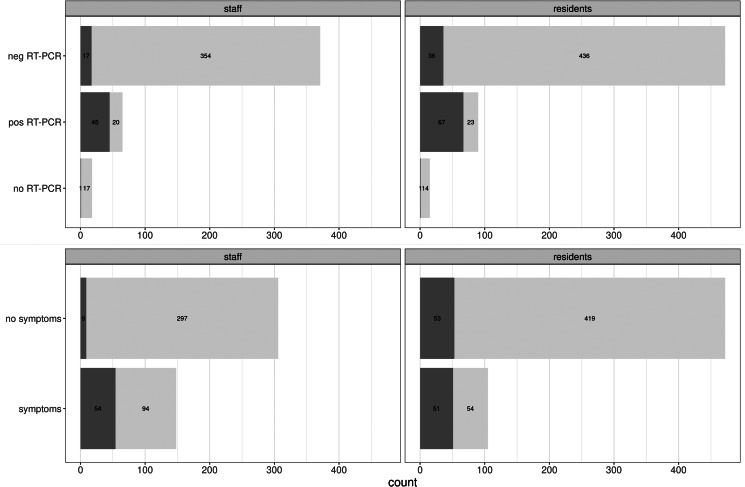
Seroprevalence in RT-PCR positive participants and symptomatic participants The bars represent the number of seropositive (dark grey) and seronegative (light grey) participants, according to the PCR test results (upper panel) or COVID-19 symptoms (lower panel), stratified for staff and residents. The numbers in the bars represent absolute numbers.

The odds for staff to have antibodies was significantly lower than for residents (OR 0.26; 95% CI 0.10–0.56). Since the interaction term between Covid-19 symptoms and the variable ‘staff/resident’ was significant (*P* < 0.05), the results were presented stratified for staff and residents (Table 4). First, the odds for those reporting a positive RT-PCR to have a positive serology result, was respectively 23 times (in residents) and 24 times (in staff) greater than for participants reporting no positive RT-PCR test. Staff with self-reported COVID-19 symptoms had almost seven times higher odds for positive serology results, while reporting Covid-19 symptoms in residents was related with only a two times higher odds for positive serology. Consequently, the relation between COVID-19 symptoms and serology is much weaker for residents.

**Table 4.** Generalised linear mixed-effects models, based on the data of those having had a PCR test, stratified for residents and staff: estimates and AIC for the null model without random effect, the null model with random effect and the final model for serology result

	Estimates (presented as OR) for the effects for serology result											
	Residents						Staff					
	Null model without random effect		Null model with random effect		Final model		Null model without random effect		Null model with random effect		Final model	
	OR	95% CI	OR	95% CI	OR	95% CI	OR	95% CI	OR	95% CI	OR	95% CI
Intercept	0.22	0.18–0.28	0.12	0.04–0.26	0.05	0.02–0.10	0.16	0.12–0.22	0.08	0.02–0.19	0.02	0.01–0.04
RT-PCR: Negative					^a^						^a^	
RT-PCR: Positive					23.36	11.17–51.61***					24.28	10.50–60.85***
No COVID-19 symptoms					^a^						^a^	
COVID-19 symptoms					2.32	1.12–4.72*					6.79	2.85–17.17***
AIC	537.4		450.0		333.7		358.6		326.3		200.1	

OR = odds ratio; CI, confidence interval; AIC = Akaike information criteria, ***P < 0.001, **P < 0.01, *P < 0.05;

a Category used as a reference category.

## Discussion

As far as we know, this is the first (large scale) study that reports the seroprevalence of SARS-CoV-2 in both staff members and residents in NH in Flanders before the start of the vaccination campaign (January 2021).

### The seroprevalence in NH in Flanders in fall 2020 was higher compared to the general population

The study demonstrated an average seroprevalence of 17.1% in residents and staff from NH across the Flemish region in the period mid-October to mid-November. This is higher than the seroprevalence of 9.2% in blood donors in the general Belgian population at the end of October 2020 [4, 5], increasing to 13.6% by November, 11th [4].

In our study, seroprevalence in residents was higher (18.9%), when compared to the numbers of the general population. Moreover, it is worth noting that this result even underestimates the true burden of the SARS-CoV-2 epidemic in NH, since we assume larger mortality for seropositive elderly (who are not included in our seroprevalence number) than for the seronegative residents [3, 8]. Comparing our results with seroprevalence reports from NH outside of Belgium is very difficult since -as far as we know- these are based on outbreak situations or convenience sampling, while our study aimed to describe a seroprevalence representative for the Flemish NH.

The seroprevalence of NH staff in our study was 14.9%, which is in line with the reported seroprevalence of 16.8% in health care workers in hospitals assessed in November 2020 (peak of the second wave in Belgium) [4]. However, it should be noticed that these results are based only on blood samples from health care workers, while our study also included staff with less or no contact with residents. Transmission in the workplace is suggested in occupations with frequent and prolonged patient contact or working in common areas. Generally, our findings add to several papers identifying SARS-CoV-2 infection as an occupational risk for healthcare workers in NH [18–22].

Mainly, our findings underscore the reports that NH are more affected by SARS-CoV-2 than the general population [3, 23]. Furthermore, in a number of participants, antibodies may already have waned. Indeed, although former research demonstrated that antibodies can be detected up to 5–7 months after infection in the elderly, we found seronegative participants reported to be RT-PCR positive. This may be explained for a number of these seronegative RT-PCR positive participants by the start of the second wave: they may be sampled before SARS-CoV-2 IgG were detectable in their blood [24]. Nevertheless, also waning of antibodies may be an explanation for this finding, since it is reported that in some individuals who develop weak antibody titres after infection, titres may become negative or are approaching baseline after ~5 months [25]. The waning of antibodies and the sudden rapid increase of the second wave makes it therefore difficult to interpret these results as specifically caused by the first wave. Further research involving repeated sampling of residents and staff would allow to get more insight into the specific antibody dynamics.

### The variation in seroprevalence between NH was high

We found a large variation in seroprevalence between NH: a number of NH were (almost) not affected, while others were hit by severe outbreaks (seroprevalences up to 45.0%). This large variation in seroprevalence was not introduced by the variation in mortality between NH: since no blood samples could be obtained from those residents who died during the first wave, one could argue that these variabilities in serology would be lesser, when mortality was taken into account. However, we noticed a positive correlation between NH seroprevalence and the percentage of COVID −19 deaths (Spearman's correlation of 0.78, *P* < 0.05). This suggests that, using seroprevalence variability between NH as a proxy for the variability in infection severity, even may be an underestimation. A study from Barros and co-workers also reported a large variation in seroprevalences (0–100%) in 15 long-term care facilities [26]. Also, in studies in which either cases or outbreaks of COVID-19 reports in NH are based on RT-PCR or self-reports, this variation between NH was seen [27, 28]. Former research demonstrated a relation between an increased transmission of the virus in the community and the number of outbreaks in NH, indicating a contribution from outside the facility [27, 29, 30]. Further, we notice a positive correlation between seroprevalences in staff and residents. This finding is also supported by the finding that the ICC of the random intercept model is 44%, indicating that the serology result of staff and residents within an NH is for a large part explained by features related to the NH. Taken together, not only individual vulnerability but also a number of factors related to the NH may play a role in the spread of SARS-CoV-2 within an NH, which occurs rather widely and rather equally among staff and residents [31, 32].

### Pre/asymptomatic persons likely contributed to transmission

Former research has suggested that transmission during the presymptomatic period or from asymptomatic persons may be an important contributing factor to rapid intrafacility transmission in NH [31–34]. In our study, respectively 14% and about 50% of the seropositive staff and residents reported no previous COVID-19 symptoms. Additional analyses further support these findings. First, the odds ratio for the relation between COVID-19 symptoms and positive serology is much lower than the odds ratio for the relation between positive RT-PCR and positive serology. Second, the relation between COVID-19 symptoms and seropositivity is weaker in residents in comparison with this relation in staff, suggesting that in elder people the typical COVID-19 symptoms are less present than in the younger staff. A meta-analysis indeed concluded that the proportion of infected people, that remained asymptomatic throughout the infection, was estimated as 20% [35]. Moreover, a number of studies suggested that a substantial part of the older residents did not have any of the typical COVID-19 symptoms [31, 33], but still developed an antibody response [36].

### RT-PCR underestimated SARS-CoV-2 infection during the first wave

Further, our results suggest that RT-PCR during the first wave underestimated the full extent of the SARS-CoV-2 exposure during outbreaks in the NH, which is in line with conclusions from former research [37, 38]. Indeed, the nasal/nasopharyngeal swabbing is suffering from the narrow window in which SARS-CoV-2 can be detected in infected persons, therefore demonstrating the limitations of this method for diagnosis. Consequently, the advice of former studies, to roll out a broad testing programme with repeated tests and to not rely only on symptoms to decide for testing [18, 33, 34, 38, 39], seems to be particularly important in NH.

Despite the particular strengths, such as the large scale and representativity at both NH and individual level with high response rates, this study is limited by the fact that we rely on self-report for both the RT-PCR test result and the COVID-19 symptoms. These questionnaire data may therefore suffer from bias, especially for the residents, for which we relied on the nurses, who filled in the questionnaires based on medical file records. Consequently, when recordings for symptoms were less accurate, this may lead to an underestimation of the residents with the typical COVID-19 symptoms. A second remark is that previous papers show that, in estimating prevalences of rare diseases, the uncertainty in the sensitivity and specificity of the used test, may result in an underestimation of the prevalence [40]. Therefore, we additionally adjusted our global prevalence by applying Bayesian inference analysis, using the data obtained in the pilot validation study [13] as prior data. This resulted in an adjusted prevalence of 16.1% (95% CI 12.3%–19.4%), which basically covers the same interval. Another important limitation is that our study suffers from a survivor bias: we have no information about the symptomatology of COVID-19 in residents who died. It is quite possible that these persons demonstrated a more typical presentation of COVID-19 than the survivors, which results in an overestimation of the fraction of asymptomatic residents in our study. This likely introduces an error in the estimates of the relation between symptoms and serology results for the residents and may be an explanation for the weaker effect between symptoms and serology in residents than in staff. A final remark is that the comparison group of blood donors, which is mainly composed of younger, healthy people with higher socioeconomic status may provide an underestimation of the Belgian SARS-CoV2 prevalence [41].

## Conclusion

In conclusion, this study revealed a seroprevalence of 17.1% residents and staff from 20 NH in Flanders, demonstrating that NH were more hit by the SARS-CoV-2 pandemic than the general population. Further, we noticed large differences between NH, suggesting that some NH may be more vulnerable than others. Finally, the results from the model suggest that broad and quick testing is needed in these facilities.

## Data Availability

Data are available at https://github.com/HeidiJanssens/SARS-CoV-2-Liantis-study
